# Non-linear connections between maternal hemoglobin during the third trimester of pregnancy and birth weight outcomes in full-term newborns: Estimating the breakpoints

**DOI:** 10.3389/fnut.2022.1031781

**Published:** 2022-12-22

**Authors:** Guilan Xie, Ruiqi Wang, Boxing Zhang, Landi Sun, Wanwan Xiang, Mengmeng Xu, Sijing Zhu, Leqian Guo, Xu Xu, Wenfang Yang

**Affiliations:** ^1^Department of Obstetrics and Gynecology, Maternal and Child Health Center, The First Affiliated Hospital of Xi’an Jiaotong University, Xi’an, Shaanxi, China; ^2^School of Public Health, Xi’an Jiaotong University Health Science Center, Xi’an, Shaanxi, China; ^3^National Medical Center Office, The First Affiliated Hospital of Xi’an Jiaotong University, Xi’an, Shaanxi, China

**Keywords:** hemoglobin, breakpoints, piecewise linear regression model, birth weight outcomes, non-linear connections

## Abstract

**Objective:**

Anemia is still an unfinished global health problem, and adverse birth weight outcomes have everlasting influences on the health of later life. However, the non-linear connections and breakpoints of maternal hemoglobin with birth weight outcomes are still needed to be further elucidated. We aimed to reveal the non-linear connections between maternal hemoglobin during the third trimester of pregnancy and birth weight, low birth weight (LBW), macrosomia, small for gestational age (SGA), and large for gestational age (LGA) in full-term newborns and elucidate the breakpoints of the connections.

**Methods:**

A total of 11,411 singletons, full-term, and live newborns, whose mothers conducted the examination of hemoglobin concentration before delivery, were included in this study. A generalized additive model was used to identify and visualize the non-linear connections between maternal hemoglobin and birth weight outcomes. Piecewise linear regression model was adopted to estimate the breakpoints of the connections and report the non-linear connections in detail.

**Results:**

There were inverted “U”-shaped exposure–response connections between maternal hemoglobin concentration and birth weight and the risk of macrosomia. There was an increasing trend of the risk of LBW and a decreased trend of LGA with the increase in maternal hemoglobin concentration. The breakpoints of maternal hemoglobin for birth weight were 100 and 138 g/L, and those for SGA were 97 and 138 g/L. The breakpoints of maternal hemoglobin were 119 g/L for LBW, 105 g/L for macrosomia, and 106 g/L for LGA. When maternal hemoglobin concentration ranged from 100 to 138 g/L, maternal hemoglobin concentration increased per 1 g/L, and birth weight significantly decreased by 2.58 g (95% CI: –3.33, –1.83). When maternal hemoglobin concentration ranged from 97 to 138 g/L, maternal hemoglobin concentration increased per 1 g/L, and the risk of SGA significantly increased by 2% (95% CI: 1%, 3%). When maternal hemoglobin concentration was equal to or lower than 119 g/L, maternal hemoglobin concentration increased per 1 g/L, and the risk of LBW significantly increased by 3% (95% CI: 0%, 5%). When maternal hemoglobin concentration was higher than the breakpoints, the risks of macrosomia (OR = 0.99, 95% CI: 0.98, 0.99) and LGA (OR = 0.99, 95% CI: 0.98, 1.00) declined as the increase of maternal hemoglobin concentration.

**Conclusions:**

There were non-linear connections between maternal hemoglobin and birth weight outcomes, and there are breakpoints in the connections. Cost-effective interventions targeting pregnant women in the prevention of abnormal maternal hemoglobin concentration should be taken to reduce the incidence of adverse birth weight outcomes.

## Introduction

Anemia in pregnancy is characterized by reduced maternal hemoglobin (Hb) concentration during pregnancy, which is one of the most common pregnancy complications. Although many countries have been devoted to attenuating the prevalence of anemia in pregnancy in the past decades by providing iron supplementation for pregnant women, it is still an unfinished global health concern. Approximately 32.4 million pregnant women are suffering from anemia in the world, and the prevalence of anemia in pregnancy is 22% in China (1). The mainstream diagnostic standard for anemia in pregnancy is that Hb concentration is lower than 110 g/L at any period during pregnancy, which is put forward by the World Health Organization (WHO), and it is beneficial to compare the prevalence of anemia in pregnancy across countries and territories (2). Given that Hb concentration varies with altitude, dietary patterns, iron supplements, smoking, and environment, the aforementioned diagnostic standard may be not suitable for all conditions (3–5). For example, US Centers for Disease Control and Prevention proposes the dynamic diagnostic standard for anemia in pregnancy, and the cutoff value is of Hb concentration for the diagnosis of anemia in pregnancy are 110, 105, and 110 g/L in the first, second, and third trimesters, respectively (6). However, some studies conducted in developing countries considered Hb concentration lower than 100 g/L as anemia in pregnancy (7, 8).

Neonatal birth weight can mirror the fetal intrauterine growth situations, and adverse birth weight outcomes are assumed to be of lifelong health effects, increasing the risks of neurodevelopmental disorders in childhood, earlier pubertal onset, and chronic non-communicable diseases in adulthood (9–11). Maternal Hb functions as the oxygen-carrying and nutrient exchanging medium, which is key for the growth of the fetus (12). Although the first and second trimesters can provide more opportunities to intervene with maternal hemoglobin, fetus grow rapidly in the third trimester, and the needs for energy and nutrition in the third trimester are far more than those of the first and second trimesters (13). Plasma volume expands with the growing demand of the placenta and fetus, while the uncoordinatedly increased number of red blood cells compared to plasma volume expansion can lead to hemodilution at the gestational age of 32–34 weeks (12). Altered maternal Hb concentration may influence the morphology and function of the placenta, subsequently influencing the growth and development of the fetus (14). Low hemoglobin concentration results in insufficient oxygen and nutrients for fetal growth, causing the fetus to fail to reach its full growth potential (15). However, high hemoglobin concentration reflects the increased blood viscosity and decreased efficiency of material exchange in the placenta, finally resulting in fetal intrauterine growth restriction (16). Thus, we put forward a hypothesis that there might be non-linear connections and breakpoints of maternal hemoglobin concentration in the third trimester and neonatal birth weight outcomes. Mounting findings have revealed the close linear connections of anemia in pregnancy with low birth weight (LBW) and small for gestational age (SGA) (17–19). Scattered studies have categorized maternal Hb concentration into different levels to reveal the changing trends for the risks of LBW in response to the increased maternal Hb levels (20, 21). However, there are still knowledge gaps in the non-linear connections and breakpoints of maternal Hb concentration and birth weight outcomes being warranted to be recognized.

In this study, we sought to verify the non-linear connections between maternal hemoglobin during the third trimester of pregnancy and birth weight, LBW, macrosomia, SGA, and large for gestational age (LGA) in full-term newborns and further estimate the breakpoints. These findings can enhance the attention to maternal Hb level during pregnancy and aid obstetricians to take timely actions to cope with the aberrant maternal Hb concentration, so as to facilitate fetal growth and development.

## Materials and methods

### Study population

Neonates who were born in the First Affiliated Hospital of Xi’an Jiaotong University from 2015 to 2019 were recruited. The clinical characteristics were extracted from the electronic medical records. Neonates were included for (1) singleton and live newborns; (2) full-term neonates with the gestational age of 37–42 weeks; (3) with the examination result of maternal Hb concentration within 7 days before delivery; and (4) maternal age was between 20 and 45 years old. We excluded those with missing information, whose mothers were with kidney diseases or hematological cancers influencing the hemoglobin concentration. This study was permitted by the Medical Ethics Committee of the First Affiliated Hospital of Xi’an Jiaotong University (No. XJTU1AF2020LSK-261).

### Measurement of maternal hemoglobin and neonatal birth weight outcomes

The collection of maternal venous blood samples was conducted before they were delivered. Then, the blood samples were detected in the department of clinical laboratory within 2 h after collection. Professional laboratorians detected the hemoglobin concentration of maternal venous blood samples with standard procedures. Newborns without clothes were placed on the electronic scale within 1 h after birth by professional midwives to evaluate their birth weight. The precision of the electronic scale was 10 g. Neonates were categorized into LBW, appropriate birth weight (ABW), and macrosomia according to the birth weight in grams of < 2,500 g, 2,500–3,999 g, and ≥ 4,000 g, and neonates were categorized into SGA, appropriate for gestational age (AGA), and LGA according to the birth weight in percentiles of < 10th, 10th–90th, and > 90th (22). The Chinese neonatal birth weight curve for different gestational ages was used as the standard of sex-specific birth weight for gestational age (23).

### Covariates

Pregnant women with low social status tend to have less access to healthcare and take irregular prenatal visits, which are related to the increased risks of adverse pregnancy outcomes (24). The quality of a woman’s eggs decreases with the increase in age and subsequently leads to suboptimal quality of embryo (25). Hypertensive disorders of pregnancy might cause arteries to spasm, impair the function of the placenta, prevent the fetus from getting oxygen and nutrients from the placenta, and even induce adverse birth outcomes (26). In addition, pregnant women with gestational diabetes mellitus (GDM) have higher risks of macrosomia, LGA, and cesarean section (27). The fetal growth trajectories show that there are sex and gestational week disparities for neonatal birth weight (23). The multiparas always have advanced age and higher risks of gestational complications. Thus, the sociodemographic covariates directly or indirectly related to neonatal birth weight were considered as the covariates in this study, including maternal age (years old), gestational weight gain (GWG), occupation (farmers/workers/others/none), ethnicity (Han/minorities), educational level (< 9 years/10–12 years/ > 12 years), parity (primipara/multipara), delivery mode (vaginal/cesarean), hypertensive disorders of pregnancy (yes/no), GDM (yes/no), gestational age (weeks), and neonatal gender (male/female). Gestational age was the period calculated from the last menstrual period to the birth date. The relationships between maternal Hb concentration, birth weight outcomes, and covariates were displayed with the directed acyclic graph in DAGitty,^1^ as shown in Supplementary Figure 1.

### Statistical analyses

Descriptive analysis was used to outline the distributions of basic characteristics. Means and standard deviations were adopted to display the continuous variables, and frequencies and proportions were utilized to present the category variables. A generalized additive model was used to identify the non-linear connections between maternal hemoglobin and birth weight outcomes after adjusting for sociodemographic covariates. Piecewise linear regression model was applied to find the breakpoints of the non-linear connections and verify the non-linear connections in detail. Furthermore, the continuous maternal hemoglobin concentrations were divided into subgroups according to the breakpoints observed in the piecewise linear regression model. In each subgroup of maternal hemoglobin concentration, the generalized linear model was adopted to elaborate the linear connection between maternal hemoglobin and neonatal birth weight outcomes. In addition, the interaction term of maternal Hb concentration × neonatal gender was added to investigate the interactive effect of maternal Hb concentration and neonatal gender on birth weight outcomes. The “mgcv” and “segmented” packages of R 4.1.2^2^ were used in this study, and the *p*-value < 0.05 was used to determine statistical significance.

## Results

### Descriptive characteristics

A total of 11,411 singleton, full-term, and live newborns were included in this study. The distributions of descriptive characteristics of the study population are shown in Table 1. Among them, there were 233 cases of LBW (2.0%), 676 cases of macrosomia (5.9%), 821 cases of SGA (7.2%), and 1,006 cases of LGA (8.8%). The means and standards of maternal age, GWG, and gestational age in total neonates were 30.0 ± 3.9 years, 15.2 ± 4.5 kg, and 39.5 ± 1.1 weeks, respectively. The means of GWG for macrosomia and LGA were higher than those for LBW, ABW, SGA, and AGA. Most mothers were with other occupations, Han ethnicity, and received > 12 years’ education. The proportion of multipara was almost half of the proportion of the primipara. However, the proportion of vaginal delivery was slightly higher than that of cesarean delivery. The prevalence of hypertensive disorders of pregnancy was higher in LBW and SGA, but the prevalence of GDM was higher in macrosomia and LGA. The means and standards of maternal hemoglobin and neonatal birth weight were 118.1 ± 13.8 g/L and 3,337.8 ± 416.6 g, respectively. For LBW, the proportion of female subjects surpassed that of male subjects, while the reverse applied for macrosomia.

**TABLE 1:** Descriptive characteristics of the study population (*n* = 11,411).

Variables	Total (*n* = 11,411)	Birth weight in grams			Birth weight in percentiles		
		LBW (*n* = 233)	ABW (*n* = 10,502)	Macrosomia (*n* = 676)	SGA (*n* = 821)	AGA (*n* = 9,584)	LGA (*n* = 1,006)
**Maternal characteristics**							
Age, years old	30.0 ± 3.9	29.5 ± 4.2	30.0 ± 3.9	30.0 ± 3.9	29.4 ± 4.0	30.0 ± 3.9	30.5 ± 4.2
GWG, kg	15.2 ± 4.5	13.5 ± 4.7	15.1 ± 4.4	17.2 ± 5.1	13.9 ± 4.4	15.2 ± 4.4	16.9 ± 5.1
**Occupation**							
Farmer	382 (3.4)	17 (7.3)	354 (3.4)	11 (1.6)	46 (5.6)	312 (3.3)	24 (2.4)
Worker	2,549 (22.3)	44 (18.9)	2,324 (22.1)	181 (26.8)	148 (18.0)	2,152 (22.4)	249 (24.8)
Others	7,015 (61.5)	135 (57.9)	6,472 (61.6)	408 (60.4)	517 (63.0)	5,884 (61.4)	614 (61.0)
None	1,465 (12.8)	37 (15.9)	1,352 (12.9)	76 (11.2)	110 (13.4)	1,236 (12.9)	119 (11.8)
**Ethnicity**							
Han	11,332 (99.3)	232 (99.6)	10,431 (99.3)	669 (99.0)	817 (99.5)	9,520 (99.3)	995 (98.9)
Minorities	79 (0.7)	1 (0.4)	71 (0.7)	7 (1.0)	4 (0.5)	64 (0.7)	11 (1.1)
**Educational level**							
≤ 9 years	947 (8.3)	42 (18.0)	860 (8.2)	45 (6.7)	99 (12.1)	772 (8.1)	76 (7.6)
10–12 years	976 (8.6)	27 (11.6)	898 (8.5)	51 (7.5)	79 (9.6)	810 (8.4)	87 (8.6)
> 12 years	9,488 (83.1)	164 (70.4)	8,744 (83.3)	580 (85.8)	643 (78.3)	8,002 (83.5)	843 (83.8)
**Parity**							
Primipara	7,638 (66.9)	158 (67.8)	7,014 (66.8)	466 (68.9)	608 (74.1)	6,399 (66.8)	631 (62.7)
Multipara	3,773 (33.1)	75 (32.2)	3,488 (33.2)	210 (31.1)	213 (25.9)	3,185 (33.2)	375 (37.3)
**Delivery mode**							
Vagina	5,973 (52.3)	73 (31.3)	5,684 (54.1)	216 (32.0)	412 (50.2)	5,228 (54.5)	333 (33.1)
Cesarean	5,438 (47.7)	160 (68.7)	4,818 (45.9)	460 (68.0)	409 (49.8)	4,356 (45.5)	673 (66.9)
**Hypertensive disorders of pregnancy**							
Yes	570 (5.0)	45 (19.3)	496 (4.7)	29 (4.3)	91 (11.1)	423 (4.4)	56 (5.6)
No	10,841 (95.0)	188 (80.7)	10,006 (95.3)	647 (95.7)	730 (88.9)	9,161 (95.6)	950 (94.4)
**GDM**							
Yes	753 (6.6)	20 (8.6)	658 (6.3)	75 (11.1)	50 (6.1)	586 (6.1)	117 (11.6)
No	10,658 (93.4)	213 (91.4)	9,844 (93.7)	601 (88.9)	771 (93.9)	8,998 (93.9)	889 (88.4)
Hb, g/L	118.1 ± 13.8	120.3 ± 13.8	118.1 ± 13.8	116.8 ± 13.5	120.0 ± 13.9	118.1 ± 13.8	116.7 ± 13.6
Gestational age, weeks	39.5 ± 1.1	38.2 ± 1.0	39.5 ± 1.0	40.1 ± 0.9	39.3 ± 1.1	39.5 ± 1.1	39.7 ± 1.1
Birth weight, g	3,337.7 ± 416.6	2,320.7 ± 166.1	3,303.5 ± 332.8	4,219.8 ± 214.0	2,608.2 ± 222.3	3,320.3 ± 298.4	4,098.9 ± 251.1
**Gender**							
Male	5,882 (51.5)	93 (39.9)	5,342 (50.9)	447 (66.1)	392 (47.7)	4,981 (52.0)	509 (50.6)
Female	5,529 (48.5)	140 (60.1)	5,160 (49.1)	229 (33.9)	429 (52.3)	4,603 (48.0)	497 (49.4)

### Non-linear connections

**FIGURE 1:**
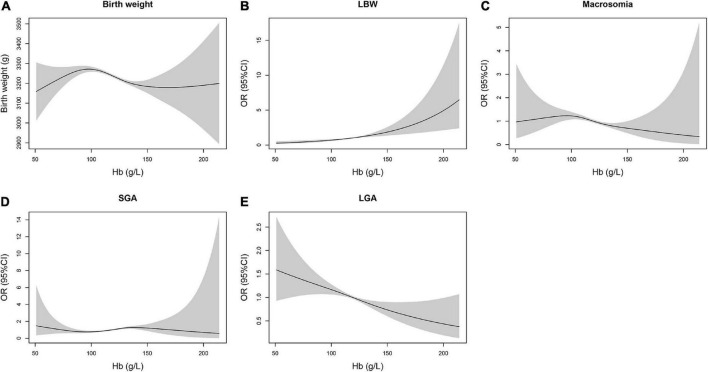
Non-linear connections between maternal hemoglobin during the third trimester of pregnancy and birth weight outcomes in neonates. **(A)** Birth weight^a^, **(B)** LBW^a^, **(C)** macrosomia^a^, **(D)** SGA^b^, and **(E)** LGA^b^. ^a^Adjusted for maternal age, occupation, ethnicity, educational level, parity, delivery mode, hypertensive disorders of pregnancy, GDM, neonatal gender, gestational age, and GWG. ^b^Adjusted for maternal age, occupation, ethnicity, educational level, parity, delivery mode, hypertensive disorders of pregnancy, GDM, and GWG.

The inverted “U”-shaped curve was discovered on the exposure–response connection between maternal hemoglobin and birth weight (Figure 1). The birth weight of newborns increased together with maternal hemoglobin concentration first and then decreased with the rise of maternal hemoglobin concentration, and the exposure–response connection was flat at high concentrations of maternal hemoglobin. The risk of LBW increased coupled with the rise of maternal hemoglobin concentration. The inverted “U”-shaped curve was also seen in the association between maternal hemoglobin and the risk of macrosomia. The fitted curve of maternal hemoglobin concentration and risk of SGA had small fluctuations. The risks of LGA decreased with the rise of maternal hemoglobin concentration.

### Breakpoints

The breakpoints of the non-linear connections between maternal hemoglobin and birth weight outcomes are shown in Table 2. The breakpoints of maternal hemoglobin for birth weight were 100 and 138 g/L. The breakpoints of maternal hemoglobin were 119 g/L for LBW, 105 g/L for macrosomia, and 106 g/L for LGA. The breakpoints of maternal hemoglobin for SGA were 97 and 138 g/L.

**TABLE 2:** Influences of per 1 g/L increase in maternal hemoglobin concentration at different segments on birth weight outcomes.

Groups	β(95% CI) or OR (95% CI)	*P*	*P* for interaction^c^
**Birth weight^a^**			
< 100 g/L	2.26(−0.73,5.26)	0.14	0.95
100–138 g/L	−2.58(−3.33,−1.83)	< 0.001	0.02
> 138 g/L	1.13(−3.35,5.60)	0.62	0.57
**LBW^a^**			
≤ 119 g/L	1.03(1.00,1.05)	0.02	0.23
> 119 g/L	1.02(0.99,1.04)	0.17	0.59
**Macrosomia^a^**			
≤ 105 g/L	1.02(0.99,1.05)	0.20	0.44
> 105 g/L	0.99(0.98,0.99)	0.001	0.81
**SGA^b^**			
< 97 g/L	0.98(0.94,1.01)	0.20	0.96
97–138 g/L	1.02(1.01,1.03)	< 0.001	0.12
> 138 g/L	0.98(0.92,1.02)	0.37	0.53
**LGA^b^**			
≤ 106 g/L	1.00(0.99,1.02)	0.71	0.46
> 106 g/L	0.99(0.98,1.00)	0.002	0.43

^a^Adjusted for maternal age, occupation, ethnicity, educational level, parity, delivery mode, hypertensive disorders of pregnancy, GDM, neonatal gender, gestational age, and GWG. ^b^Adjusted for maternal age, occupation, ethnicity, educational level, parity, delivery mode, hypertensive disorders of pregnancy, GDM, and GWG. ^c^*P* for interaction was the *p*-value for the interaction term of maternal Hb concentration × neonatal gender.

### Non-linear connections in detail

The influences of maternal hemoglobin at different segments on birth weight outcomes are shown in Table 2. When maternal hemoglobin concentration ranged from 100 to 138 g/L, maternal hemoglobin concentration increased per 1 g/L, and birth weight significantly decreased by 2.58 g (95% CI: –3.33, –1.83). However, when maternal hemoglobin concentration was lower than 100 g/L or higher than 138 g/L, birth weight increased insignificantly with the rise of maternal hemoglobin concentration. When maternal hemoglobin concentration was equal to or lower than 119 g/L, maternal hemoglobin concentration increased per 1 g/L, and the risk of LBW significantly increased by 3% (95% CI: 0%, 5%). While there was no statistically significant association between maternal hemoglobin concentration and the risk of LBW when maternal hemoglobin concentration was higher than 119 g/L (OR = 1.02, 95% CI: 0.99, 1.04). When maternal hemoglobin concentration was higher than 105 g/L, the risk of macrosomia declined with the increase in maternal hemoglobin concentration (OR = 0.99, 95% CI: 0.98, 0.99). When maternal hemoglobin concentration ranged from 97 to 138 g/L, maternal hemoglobin concentration increased per 1 g/L, and the risk of SGA significantly increased by 2% (95% CI: 1%, 3%). However, when maternal hemoglobin concentration was lower than 97 g/L or higher than 138 g/L, there was no statistically significant association between maternal hemoglobin concentration and the risk of SGA. When maternal hemoglobin concentration was higher than 106 g/L, the risk of LGA declined with the increase in maternal hemoglobin concentration (OR = 0.99, 95% CI: 0.98, 1.00). The statistically significant association between maternal hemoglobin concentration and risk of LGA was not found, when maternal hemoglobin concentration was equal to or lower than 106 g/L. In addition, there was no interaction effect of maternal hemoglobin concentration and neonatal gender on birth weight outcomes (*P* for interaction > 0.05), except for birth weight in the segment of maternal hemoglobin concentration ranging from 100 to 138 g/L (*P* for interaction = 0.02).

## Discussion

We investigated the non-linear connections between maternal hemoglobin during the third trimester of pregnancy and birth weight, LBW, macrosomia, SGA, and LGA in full-term newborns, as well as estimated the breakpoints of the non-linear connections.

The fetus grows fast during the third trimester for the rapid development of somatic cells and adipose tissues (12). Therefore, maternal hemoglobin concentration in late pregnancy is critical for fetal growth. The majority of studies have reported that anemia during pregnancy increases the probability of suboptimal birth weight (18, 28, 29). Lumbanraja et al. (18) found that the distributions of LBW were different between the anemia in the pregnancy group and the normal hemoglobin in the pregnancy group. Two systematic review and meta-analysis studies indicated that maternal anemia was significantly linked to the enhanced risk of LBW but not in SGA (28, 29). Several studies have explored the linear connection between maternal hemoglobin concentration during the third trimester and birth weight (5, 30). Jwa et al. (30) applied multiple regression analysis and found that per 1 g/dL increase in Hb in late pregnancy reduced birth weight by 73.2 g (95% CI: –90.0 to –56.4 g). Nahum et al. (5) predicted term birth weight with a regression equation on the basis of maternal hemoglobin concentration, effects on birth weight (g) = 1,020–[88 × maternal 3rd-trimester hemoglobin concentration (g/dL)]. Limited studies explored the non-linear connection between maternal hemoglobin concentration and LBW (31, 32). A prospective population-based study in rural Bangladesh showed a U-shaped relationship between maternal hemoglobin concentration with the risk of LBW, with those with high hemoglobin concentration having a 128% increased risk of LBW compared with those with mild anemia (31). Another study collected data from Pakistan and India, divided maternal hemoglobin concentration into seven levels, and found that with the reference of normal range of maternal hemoglobin concentration (11.0–12.9 g/dL), lower maternal hemoglobin levels were related to higher risks of LBW (32). A cluster-randomized controlled trial study in rural areas of Northwest China also fitted the inversed “U”-shaped curve between maternal hemoglobin concentration and birth weight (33). In this study, maternal hemoglobin concentration and birth weight were treated as continuous variables to verify the Hb concentration varying trend of birth weight and found the inversed “U”-shaped non-linear connection between maternal hemoglobin and birth weight, which was congruent with the previous studies.

The relationships between anemia in pregnancy and birth weight outcomes divert in pace with the cutoff values of maternal hemoglobin concentration (34, 35). A meta-analysis pooling the results of 95 studies suggested that low maternal hemoglobin concentration was tied to the increased odds ratios of LBW and SGA, and the connections became stronger when the cutoff values of abnormal maternal hemoglobin concentration were lower (34). Those with low maternal Hb had higher odds ratios of LBW and SGA when low maternal Hb concentration was deemed as Hb ≤ 100 g/L than when low maternal Hb concentration was deemed as Hb ≤ 110 g/L. Shinar et al. (35) applied receiver operation characteristics curves in 651 pregnant women and held that the hemoglobin threshold of anemia in pregnancy should be redefined as 100 g/L in singleton pregnancies. In this study, the breakpoints of the non-linear connection between maternal hemoglobin and birth weight outcomes were estimated to range from 97 to 138 g/L, which were incongruent with the widely used cutoff value of anemia in pregnancy put forward by WHO (2). Indeed, hemoglobin concentration is influenced by multi-facet factors (4, 36–38). Hemoglobin concentration declines with the increase in altitude in response to the declined blood oxygen saturation in high altitude places (36). Carbon monoxide in tobacco smoke can promote the formation of carboxyhemoglobin in red blood cells and induce a compensatory increase in hemoglobin (37). In addition, lower hemoglobin concentrations are associated with low socioeconomic status, imbalanced dietary patterns, multiparity, and high concentration exposure to air pollution (4, 38). Thus, the cutoff value of hemoglobin concentration for anemia in pregnancy should be reevaluated according to different conditions, so as to avoid the misclassification of anemia. The breakpoints of the non-linear connections between maternal hemoglobin and birth weight outcomes in full-term newborns are of great significance to instruct healthcare providers in intervening timely and properly to eliminate the negative effects of inappropriate maternal hemoglobin concentration, so as to enable the fetus to fulfill optimal growth and development.

Both extremely low and extremely high maternal hemoglobin concentrations jeopardize neonatal birth weight (33). Mild anemia in pregnancy might link to adequate blood volume expansion, which is conducive to placental blood circulation (39). During pregnancy, the fetus grows with the deprivation of nutrition from its mothers, and nutrients are preferentially transferred to the fetus (40). That is why the optimal neonatal birth weight occurs when pregnant women are with mild anemia in pregnancy. However, moderate-to-severe anemia in pregnancy may influence placental angiogenesis and contribute to the decreased oxygen-carrying capacity and insufficient nutrient supply, ultimately resulting in reduced neonatal birth weight (41, 42). A study including 1,986 singleton pregnant women indicated that Hb concentration in late pregnancy was inversely correlated with placental weight and placental ratio (30). Conversely, extremely high maternal hemoglobin concentration manifests inadequate blood volume expansion and increased blood viscosity, and the attenuated hemodynamic velocity is associated with the declined efficiency of material exchange in the placenta (43, 44). In addition, the extremely high maternal hemoglobin concentration might cause blood vessel blockage in the placenta, induce intrauterine oxidative stress, and further lead to intrauterine growth restriction (45). The birth weight of male newborns is heavier than that of female newborns due to androgen action (46). In comparison with the female fetus, the birth weight of a male fetus is more subject to maternal nutritional status for the gender-different intrauterine physical adaptations (47). However, the interactive effect of maternal hemoglobin and neonatal gender on birth weight was only found in the middle range of maternal hemoglobin concentration.

There were several strengths in this study. First, non-linear connections between maternal hemoglobin during the third trimester of pregnancy and birth weight outcomes in full-term newborns were manifested, which expanded the knowledge of exposure–response relationships between maternal hemoglobin and birth weight outcomes. Second, breakpoints of the non-linear connections were innovatively estimated with a piecewise linear regression model, which was beneficial to redefine the cutoff values of hemoglobin concentration for anemia in pregnancy and develop targeted intervention strategies. Meanwhile, there were still some limitations that need to be mentioned. First, anemia in pregnancy is mostly attributed to iron deficiency (48). WHO recommends daily 30–60 mg of elemental iron supplementation and 400 μg folic acid supplementation throughout pregnancy for the prevention of anemia in pregnancy, and the hemoglobin concentration in pregnant women with anemia should be corrected until normal (49). Therefore, failing to adjust the usage of iron supplementation in pregnancy was a critical limitation in this study. Second, imbalanced dietary patterns and insufficient consumption of high-quality protein link to increased risk of anemia in pregnancy (50). However, the relevant information on diet patterns and protein consumption was not assessable for this retrospective study. Third, smoking has direct toxic impacts on neonatal birth weight and indirect impacts mediated by altered hemoglobin concentration (51). Although exposure to smoking was not adjusted as the confounding variable, smoking was scarce in Chinese pregnant women. Forth, owing to the missing data on maternal height in this retrospective birth cohort study, maternal body mass index was unable to be adjusted as one of the confounders, which was closely related to neonatal birth weight outcomes (52). However, gestational weight gain during pregnancy was adjusted, which could embody the maternal nutritional status during pregnancy to some extent (53). Thus, the findings of this study should be extrapolated with caution, and further multi-center studies were still warranted.

## Conclusion

Taken together, we revealed the non-linear connections between maternal hemoglobin during the third trimester of pregnancy and birth weight outcomes in full-term newborns and figured out the breakpoints of the dose–response connections. Our findings can drive cost-effective interventions targeting pregnant women in the prevention of abnormal maternal hemoglobin concentration, consequently, reducing the incidence of adverse birth weight outcomes.

## Data availability statement

Data described in this manuscript will be made available upon reasonable request.

## Ethics statement

The studies involving human participants used anonymized data, and were reviewed and approved by the Medical Ethics Committee of the First Affiliated Hospital of Xi’an Jiaotong University (No. XJTU1AF2020LSK-261). Written informed consent for participation was not required for this study in accordance with the national legislation and the institutional requirements.

## Author contributions

GX and RW: conceptualization, investigation, methodology, writing—original draft, and writing—review and editing. BZ, LS, WX, MX, and SZ: investigation and writing—review and editing. LG and XX: writing—review and editing. WY: conceptualization, funding acquisition, supervision, and writing—review and editing. All authors have read and approved the final manuscript.

## Footnotes

1
http://www.dagitty.net/

2
https://www.r-project.org/
