# Supplementary material for: Non-linear connections between maternal hemoglobin during the third trimester of pregnancy and birth weight outcomes in full-term newborns: Estimating the breakpoints
Source: Front Nutr. 2022 Dec 22;9:1031781. doi: 10.3389/fnut.2022.1031781 (PMC9815559; doi:10.3389/fnut.2022.1031781)
Supplement: Supplementary file 1 [file Data_Sheet_1.PDF]

Supplementary Material

Non-linear connections between maternal hemoglobin during the third trimester of pregnancy and birth weight outcomes in full-term newborns: estimating the breakpoints

Guilan Xie[1,2†], Ruiqi Wang[1,2†], Boxing Zhang[1,2], Landi Sun[1,2], Wanwan Xiang[1,2], Mengmeng Xu[1], Sijing Zhu[1], Leqian Guo[1], Xu Xu[3], Wenfang Yang[1*]

[1]Department of Obstetrics and Gynecology, Maternal & Child Health Center, The First Affiliated Hospital of Xi'an Jiaotong University, Xi'an, Shaanxi Province, People's Republic of China

[2]School of Public Health, Xi'an Jiaotong University Health Science Center, Xi'an, Shaanxi Province, People's Republic of China

[3]National Medical Center Office, The First Affiliated Hospital of Xi'an Jiaotong University, Xi'an, Shaanxi Province, People's Republic of China

[†]These authors contributed equally to this work and share first authorship.

*** Correspondence:**
Wenfang Yang
wenfang.yang@xjtu.edu.cn

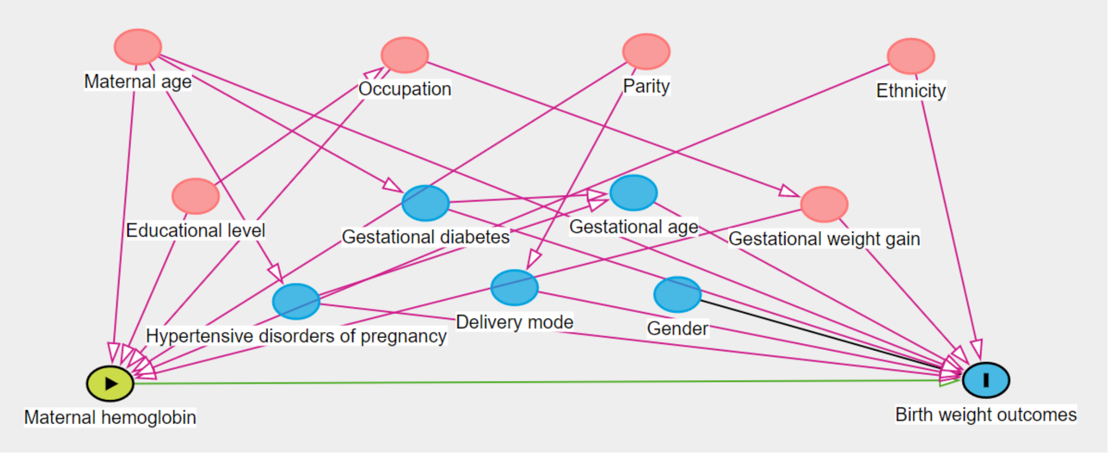

FIGURE S1. Directed acyclic graph for the relationships between maternal Hb concentration, birth weight outcomes, and covariates.
